# Lung abscess with chronic cough secondary to xanthogranulomatous pyelonephritis: A rare case report

**DOI:** 10.1097/MD.0000000000033787

**Published:** 2023-05-12

**Authors:** Pai-Yu Cheng, Yi-You Huang, Fu-Shan Jaw, Shiu-Dong Chung

**Affiliations:** a Department of Biomedical Engineering, College of Medicine and College of Engineering, National Taiwan University, Taipei, Taiwan; b Divisions of Urology, Department of Surgery, Far Eastern Memorial Hospital, Taipei, Taiwan; c Department of Nursing, College of Healthcare & Management, Asia Eastern University of Science and Technology, Taipei, Taiwan; d General Education Center, Eastern University of Science and Technology, Taipei, Taiwan.

**Keywords:** case report, chronic cough, lung abscess, radical nephrectomy, xanthogranulomatous pyelonephritis

## Abstract

Xanthogranulomatous pyelonephritis (XGPN) is a form of chronic pyelonephritis caused by chronic calculus obstruction and bacterial infection, leading to the destruction of the renal parenchyma and calyces. Conservative treatment is usually not sufficient, and surgical intervention is still the main curative approach. XGPN with transdiaphragmatic extension and lung abscess formation is a rare condition.

**Patient concerns:**

We report a 64-year-old woman who presented with persistent productive cough.

**Diagnoses:**

Lung abscess secondary to XPGN. Both nephrostomy urine and sputum cultures showed *Proteus mirabilis* infection with the same antibiotic sensitivity spectrum, but blood culture was negative.

**Interventions:**

Laparoscopic radical nephrectomy and prolonged antibiotic treatment.

**Outcomes:**

The lung abscess and cough gradually resolved in 1 month after nephrectomy.

**Conclusion:**

Lung abscess secondary to transdiaphragmatic extension of XGPN is rare but should be considered in patients with lower lung infections that are unresponsive to treatment, especially infections due to unusual respiratory pathogens such as *P mirabilis*.

## 1. Introduction

Xanthogranulomatous pyelonephritis (XGPN) is a chronic infection of the parenchyma and calyces of the kidney. It predominantly affects middle-aged women, and the most frequently reported clinical signs are anemia, chronic fever, and loin mass.^[1]^ XGPN with transdiaphragmatic extension and intrathoracic infection is a rare condition. We report a rare case of a lung abscess secondary to XGPN.

## 2. Case presentation

**Figure 1.**
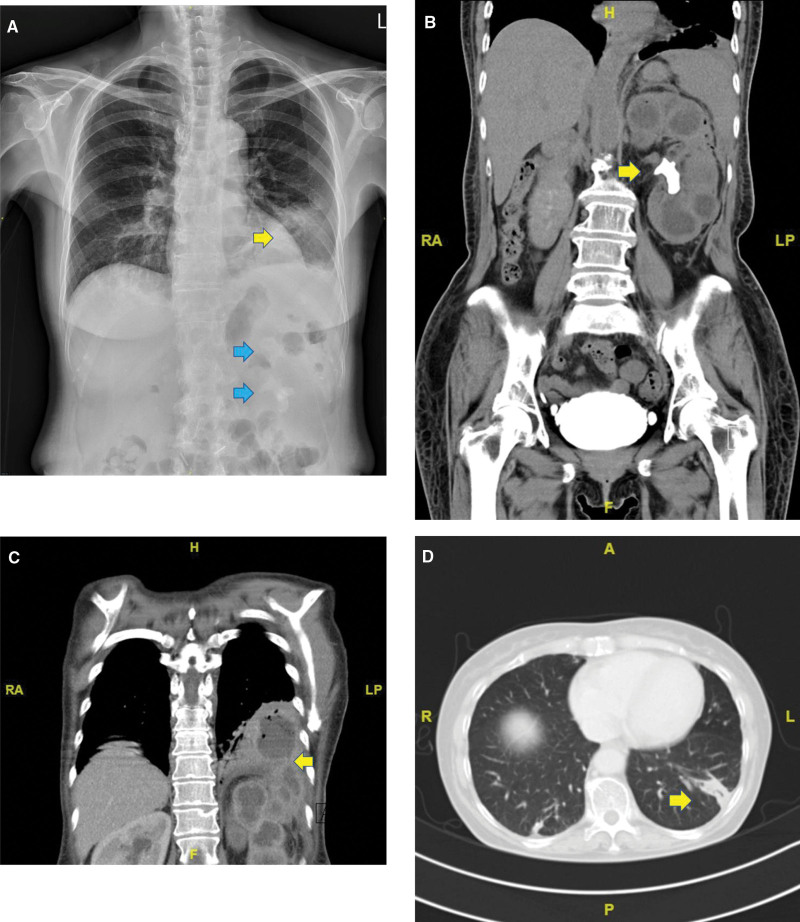
Imaging findings. (A) Chest X-ray on admission, showing left lower lung consolidation with costophrenic angle blunting (yellow arrow) and staghorn renal calculi over the left kidney (blue arrow), (B) computed tomography showing a staghorn calculus in the left kidney, with an enlarged kidney (yellow arrow), (C) computed tomography showing an abscess in the left lung and a tract of transdiaphragmatic extension (yellow arrow), and (D) computed tomography showing regression of the left lung abscess and subsegmental atelectasis 4 months after surgery (yellow arrow).

A 64-year-old woman presented with a productive cough for 2 weeks, accompanied by anorexia and weight loss. She had a history of uterine cancer that was treated with transabdominal hysterectomy. Despite receiving antibiotics provided by a local clinic, she developed progressive shortness of breath and cough; therefore, she visited our cardiovascular department for evaluation of suspected heart failure. Chest radiography showed left lower lung consolidation and costophrenic angle blunting but no cardiomegaly (Fig. 1A). Left lower lobe pneumonia was suspected and the patient was transferred to the emergency department.

A COVID-19 real-time PCR test was negative. Hematology showed bandemia (white blood cells, 7.77 × 10^3^ cells/μL; neutrophils, 74.4%; band cells, 4.1%) and anemia (hemoglobin 7.2 g/dL). Blood biochemistry revealed elevated levels of N-terminal pro-brain natriuretic peptide (925 pg/mL; normal, <125 pg/mL) and D-dimer (1.66 mg/L; normal, <0.55 mg/L), but troponin-T was within normal limits (6.5 ng/L; normal, <14 ng/L). Chest computed tomography (CT) angiography revealed a left lower lobe consolidation lesion with 4 × 3.7 cm of internal fluid accumulation and mild wall enhancement, and the patient was diagnosed with a lower lung abscess. CT also revealed a left renal staghorn stone with an enlarged kidney, dilated calyces, and thick cortex (Fig. 1B), leading to a diagnosis of XGPN. Fluid accumulation extended through the left perirenal, pararenal, and subphrenic spaces (Fig. 1C). The fluid was drained via percutaneous nephrostomy and empirical cefoperazone (2000 mg) and sulbactam (2000 mg) were administered every 12 hours. Both nephrostomy urine and sputum cultures showed *Proteus mirabilis* infection with the same antibiotic sensitivity spectrum, but blood culture was negative. A minimal amount of nephrostomy drainage was noted, and serial chest radiography revealed progressive lung opacities. Left laparoscopic radical nephrectomy was performed, and a Jackson-Pratt drain was inserted for 2 weeks. The patient had a spiking fever for 5 days after surgery, but her sputum production gradually diminished. Follow-up chest radiography showed patchy opacity and less pleural effusion. The patient was discharged 14 days after postoperative piperacillin 4000 mg and tazobactam 500 mg every 6 hours. Follow-up CT revealed resolution of the left lower lung abscess with subsegmental atelectasis (Fig. 1D).

## 3. Discussion

XGPN is a chronic kidney infection, usually secondary to renal calculi. *Proteus* is the most common organism found in XGPN, followed by *Escherichia coli* in approximately 30% of patients.^[2]^ Common presentations include flank or abdominal pain, fever, weight loss, and recurrent urinary tract infections.^[3]^

The diagnosis of XGPN is difficult because of its vague symptoms, which may mimic those of neoplasm.^[4]^ The typical features of XGPN on urography include unilateral renal enlargement, a nonfunctioning kidney, and the presence of large renal calculi. CT is widely used to diagnose and stage XGPN.^[5]^ A characteristic CT finding is an enlarged kidney with multiple low-density masses (“bear paw sign”).^[6]^ XGPN is staged according to its extension and involvement of the surrounding tissue, as described by Malek and Elder.^[1]^ Although percutaneous drainage combined with long-term antibiotic administration has been reported in a few case reports, surgical intervention is the main curative approach.^[7]^ The most common surgical procedure, especially in cases of diffuse inflammation involving adjacent organs, is open nephrectomy using a thoracoabdominal approach.^[8]^ Although laparoscopic nephrectomy is more challenging, it has been reported to be safe in patients with XGPN, although the conversion rate is high in difficult cases.^[9]^

Unusual clinical presentations described in case reports include acute peritonitis, ischemic colitis, sciatica, empyema, and nephrobronchial fistula,^[10–14]^ XGPN with transdiaphragmatic extension through erosion is rare.^[15]^ Identical bacterial species with the same drug spectrum from both nephrostomy urine and sputum cultures supported our findings. The negative blood culture suggests that the lung abscess was probably due to XGPN with local extension rather than due to hematogenous spread.

## 4. Conclusion

The diagnosis of XGPN in patients with atypical presentation is challenging. Lung abscesses secondary to transdiaphragmatic extension of the XGPN are rare, but should be considered in patients with lung abscesses that are unresponsive to treatment.

## Acknowledgements

We would like to acknowledge the support of the Far Eastern Memorial Hospital. We would also like to acknowledge the contributions of our colleagues.

## Author contributions

**Conceptualization:** Pai-Yu Cheng, Yi-You Huang, Fu-Shan Jaw, Shiu-Dong Chung.

**Data curation:** Shiu-Dong Chung.

**Investigation:** Pai-Yu Cheng.

**Resources:** Pai-Yu Cheng, Yi-You Huang, Fu-Shan Jaw.

**Software:** Pai-Yu Cheng.
